# Supplementary material for: Ubiquitous Love or Not? Animal Welfare and Animal-Informed Consent in Giant Panda Tourism
Source: Animals (Basel). 2023 Feb 17;13(4):718. doi: 10.3390/ani13040718 (PMC9952821; doi:10.3390/ani13040718)
Supplement: Supplementary file 1 [file animals-13-00718-s001.zip › animals-2158254-supplementary.pdf]

Supplementary Table S1. Domain 1: Nutrition and affects.

Negative Conditions		Positive Conditions	
Nutritional inadequacies	Negative affects	Nutritional opportunities	Positive affects
Restricted water intake<br>Excessive water intake	Thirst<br>Water intoxication	Drink correct quantities of water	Wetting/quenching pleasures of drinking
Restricted food intake	Hunger (general)<br>Hunger (salt)<br>Weakness of starvation	Eat enough food	Postprandial satiety<br>Pleasure of salt taste
Poor food quality<br>Low food variety	Malaise of malnutrition<br>Eating-related boredom	Eat a balanced diet<br>Eat a variety of foods	Pleasures of food tastes, smells, textures
Voluntary overeating	Bloated or overfull	Eat correct quantities of food	Comfort of satiety
Force-feeding, excessive energy intake	Gastrointestinal pain, nausea/malaise		Gastrointestinal comfort
Anabolic steroids for performance	Increased libido in males and testicular atrophy, and abnormal sexual behaviour in females; edema formation; epiphyseal plate closure, retards growth [a]	Anabolic steroids for normal health and recovery	Prevent muscle atrophy form disease, if used in moderation<br>Increase weight gain and appetite, and improve attitude [a]

[a] Dowling (2021)

Supplementary Table S2. Domain 2: Physical environmental conditions and affects.

Negative Conditions		Positive Conditions	
Unavoidable physical conditions	Negative affects: forms of discomfort	Enhanced physical conditions	Positive affects: forms of comfort
Close confinement, overcrowding<br>Unsuitable substrate, wet/soiled ground	Physical: general stiffness, muscle tension<br>Physical: musculoskeletal pain, skin irritation	Space for spontaneous locomotion<br>Suitable substrate, well-drained ground	Physical comfort<br><br>Physical comfort
Air pollutants:<br>NH_3, CO_2, dust, smoke	Respiratory: breathlessness, air passage irritation/pain	Fresh air dissipates contaminates	Respiratory comfort
Aversive odours	Olfactory: revulsion at foul or repellent odours	Foul smells dissipated by fresh air & good hygiene	Olfactory comfort
Thermal extremes	Thermal: chilling, dampness, overheating	Effective shelter and shade available	Thermal comfort
Loud or otherwise unpleasant noise	Auditory: impaired hearing or ear pain	Effective noise control measures are in place	Auditory comfort

Light: inappropriate intensity	Visual: eye strain due to flashing, glare or darkness	Light intensity kept at tolerable levels	Visual comfort
Monotony: ambient, physical, lighting	Malaise from unnatural constancy	Within-day environmental variability maintained	Congenial variety and predictability
Unpredictable events	Anxiety, fear, hypervigilance	Predictability achieved by established routines	Relaxation-based ease and calmness
Physical limits on rest and sleep	Exhaustion	Conditions conducive to rest and sleep	Well rested

Supplementary Table S3. Domain 3: Health conditions and affects.

Negative Conditions		Positive Conditions	
Presence of:	Negative affects	Minimal or no:	Positive affects
Injury: acute, chronic, husbandry mutilations	Pain (many types), breathlessness, debility, weakness, sickness, malaise, nausea, dizziness	Injury	Comfort of good health and functional capacity
Disease: acute, chronic		Disease	Comfort of good health and functional capacity
Functional impairment: due to limb amputation, other therapies; genetic, lung, heart, vascular, kidney, gut, neural, or other problems		Functional impairment	Comfort of good health and functional capacity
Obesity or leanness: physical and metabolic consequences	Affects of being too fat or thin, and of metabolic and pathophysiological sequelae	Extreme body condition scores	Comfort of good health and functional capacity
Poisons	Many affects due to mode of action	Poisoning	Comfort of good health and functional capacity
Poor physical fitness, muscle de-conditioning	Physical weakness and exhaustion	Poor fitness (fitness level good)	Vitality of fitness and pleasurable vigorous exercise

Supplementary Table S4. Domain 4: Behavioural interactions and affects.

INTERACTIONS WITH THE ENVIRONMENT			
Exercise of 'agency' is impeded	Negative affects	Exercise of 'agency' is promoted	Positive affects:
Invariant, barren, confined environment (ambient, physical, biotic)	Boredom, helplessness Depression, withdrawal	Varied, novel environment	Interested, pleasantly occupied

Inescapable sensory impositions<br><br>Choices markedly restricted<br><br>Environment-focussed activity constrained<br><br>Foraging drive impeded	Various combinations: startled by unexpected events, neophobia, hypervigilance, anger, frustration, negative cognitive bias	Congenial sensory inputs<br><br>Available engaging choices<br><br>Free movement<br><br>Exploration, foraging	Likes novelty, post-inhibitory rebound<br><br>Calm, in control<br><br>Engaged by activity<br><br>Energised, focussed
INTERACTIONS WITH OTHER ANIMALS			
Animal-to-animal interactive activity constrained	Loneliness, depression<br>Yearning for company<br><br>Thwarted desire to play<br>Sexual frustration<br>Thwarted hunting drive	Bonding/reaffirming bonds<br>Rearing young<br><br>Playing<br>Sexual activity<br>Hunting	Affectionate sociability<br><br>Maternal, paternal or group rewards<br><br>Excitation/playfulness<br>Sexually gratified<br>Alert engagement, highly stimulated
Significant threats<br>Limits on threat avoidance, escape or defensive activity<br>Limitations on sleep/rest	Anger, anxiety, fear, panic, insecurity, neophobia<br><br>Exhaustion	Absence of threats<br>Using refuges, retreat or defensive attack<br><br>Sleep/rest sufficient	Secure, protected, confident<br><br>Energised, refreshed; post-inhibitory rebound
INTERACTIONS WITH HUMANS			
Negative human attributes and behaviour	Animal behaviours and negative affects	Positive human attributes and behaviour	Animal behaviours and positive affects
Attitude: uncertain, fearful, indifferent, insensitive, impatient, oppressive, belligerent, domineering, callous, cruel, vindictive	Behaviours (e.g.,): long flight distance, hypervigilant, attack/flight, hyper-reactive, escape avoidance, freezing, cowering, appeasing, withdrawn, non-compliant	Attitude: confident, caring, sensitive, patient, kind, empathetic	Behaviours: short flight distance, calm alertness, at ease with imposed hands-off or hands-on contact, compliantly responsive, explores novel events, seeks contact, variably bonded with humans
Voice: hesitant, angry, loud, shouting		Voice: confident, calm, dear, encouraging, pleasantly rhythmic	
Aptitude: inexperienced, unskilled, untrained, unqualified		Aptitude: experienced, skilled, trained, qualified	
Handling/controlling: erratic, rough (slap, hit, kick, grab, poke, beat,	Affects: anxiety, fear, panic, terror, neophobia; insecurity,	Handling/controlling: skillful, gentle (stroke, touch, push, guide);	Affects: calm, confident, at ease, feels in control; enjoys variety;

whip); excessively forceful, violent; punishment-focussed; more negative pressure than is needed for training objective	confusion, uncertainty, persistent unease; helplessness; pain from injuries; negative cognitive bias	firm, temperate, restrained; reward-focussed; mimics allogrooming by conspecifics; using subtle pressure cues, secondary reinforcers and timely release of aversive stimuli	finds being bonded with humans rewarding

Source: Adapted from Mellor et al. (2020) for all tables
